# Specialist training in general practice: Developments in social-legislation-based support – a data-driven introduction

**DOI:** 10.3205/zma001707

**Published:** 2024-11-15

**Authors:** Simon Rass, Charlotte Weber, Bernhard Gibis

**Affiliations:** 1 Kassenärztliche Bundesvereinigung, Dezernat Sicherstellung und Versorgungsstruktur, Abteilung Sicherstellung, Berlin, Germany

**Keywords:** physicians, family – supply & distribution, primary health care – statistics & numerical data, primary health care – trends, federal government, education, medical, graduate – economics, training support – economics, health manpower – statistics & numerical data, health manpower – trends, health policy, health services needs and demand – trends, insurance, health, reimbursement, physicians, family – education, population dynamics, humans, Germany

## Abstract

**Aims:**

Comprehensive provision of general healthcare (i.e. primary care) within the populace is contingent on there being enough general practitioners (GPs) in proximity to patients. It is no longer the case that vacated allocated positions for primary-care physicians are being filled in all regions. Support for specialist training in general medical practice is one of the measures intended to ensure provision of GP services. This analytical project aims to make a data-driven contribution to gauging the impact of such support on primary care in Germany, while also delivering pointers for further research.

**Methods:**

On the basis of routinely collected data, the history of such support was examined in detail for all practice-based, statutory health insurance (SHI)-accredited GPs during the period 2016–2022. In the analysis, GPs were broken down by whether they took up full-time or part-time roles, self-employed or salaried roles, and roles in a practice or in an ambulatory healthcare centre (MVZ).

**Results:**

During the period under review, the proportion of those who have both recently commenced work as SHI-accredited GPs and had previously used support for their specialist training, increased from 57% to 81%. The total number of new GPs (headcount) rose from 1,590 to 1,955. Results indicate that those who had availed themselves of this support take up self-employed and full-time roles more often than those who had not.

**Conclusions:**

Both take-up of support for specialist training, and the number of new GPs, increased markedly during the period under review. The data does not indicate any causal links. However, these results could form a jumping-off point for further research (in general) into support for specialist training, and (in particular) into how this may impact these individuals’ subsequent work roles.

## Zusammenfassung

**Zielsetzung:**

Eine umfassende allgemeinmedizinische bzw. hausärztliche Versorgung der Bevölkerung setzt eine hinreichende Anzahl von Allgemeinärzt*innen in räumlicher Nähe zu den zu versorgenden Patient*innen voraus. Die Nachbesetzung freiwerdender Hausarztsitze gelingt nicht mehr in allen Regionen. Die Förderung der Weiterbildung in Allgemeinmedizin ist eine der Maßnahmen, mit denen das allgemeinmedizinische Leistungsangebot gesichert werden soll. Das Auswertungsprojekt soll einen datengestützten Beitrag zur Einschätzung der Auswirkungen der Förderung auf die hausärztliche Versorgung in Deutschland bieten und Hinweise für weitere Forschung geben.

**Methodik:**

Auf Basis von Routinedaten wird die Förderhistorie aller in den Jahren 2016–2022 in der vertragsärztlichen Versorgung niedergelassenen Allgemeinärzt*innen näher betrachtet. Ausgewertet wird auch die Tätigkeitsaufnahme in Voll-/Teilzeit, in Selbstständigkeit oder Anstellung und in Praxen oder Medizinischen Versorgungszentren.

**Ergebnisse:**

Im untersuchten Zeitraum ist der Anteil der neu in der vertragsärztlichen Versorgung tätigen Allgemeinärzt*innen, die eine Weiterbildungsförderung in Anspruch genommen haben, von 57% auf 81% gestiegen, die Anzahl der Neuzugänge aller Allgemeinärzt*innen (Personen) von 1.590 auf 1.955. Die Ergebnisse geben Hinweise darauf, dass an der Förderung Teilnehmende offenbar häufiger in Selbstständigkeit und Vollzeit ihre Tätigkeit aufnehmen als solche, deren Weiterbildung ungefördert war.

**Schlussfolgerungen:**

Sowohl die Inanspruchnahme der Weiterbildungsförderung als auch die Anzahl der Neuzugänge von Allgemeinärzt*innen sind im Beobachtungszeitraum deutlich angestiegen. Kausale Zusammenhänge lassen sich anhand der Daten nicht herstellen. Die Ergebnisse können jedoch Ausgangspunkt einer weitergehenden Beforschung der Weiterbildungsförderung im Allgemeinen und der Frage der Auswirkungen auf die spätere Tätigkeit im Besonderen werden.

## 1. Introduction

Sufficiently qualified and available skilled professionals, who take up work where patients require medical treatment, form the cornerstone of each and every healthcare system. In order that these population-based needs for healthcare provision (including those for general medical care) can be met everywhere, various balances need to be struck. Both foundational and specialist training must be influenced by policy such that enough prospective doctors pursue this speciality, which – as sources such as leading healthcare advisory body “SVR Gesundheit & Pflege” has clearly stated [1] – is of vital importance to the functioning of the healthcare system. It is fundamentally important that these healthcare professionals have their workplace locally in close proximity to the need, so that there is full access to primary care.

A balancing act of this nature cannot be achieved without aspects that provide a policy framework for guidance, such as the requirements-planning directive provided by the Federal Joint Committee (G-BA) [2], [3]. This framework does not, however, include the entire medical career path beginning with foundational and specialist training, but rather comes into play when a physician starts work as a specialist in a particular region (Note: for the purposes of this article, general practitioners (GPs) are considered to have specialist status as specialists in general practice.). 

One option would be to develop the regulatory framework in a restrictive manner, for example by exerting management control over specialist training through application of quotas for posts; another could involve incentive models that permit freedom in career choices and professional practice. Support for specialist training in the form of an incentive model put in place by social legislation (Volume V of the German Social Insurance Code (SGB V) [4], has been in existence since 1999; its focus is on general medical practice, and several billion euros have been spent on this since its introduction [5]. Although support for specialist training is evaluated on an annual basis, there is a lack of more in-depth accompanying studies, which can be viewed under the broader heading of educational research. 

Aspects of interest here include not only evaluation of the communication of medical knowledge and skills, but also how we assess the effectiveness of incentive models aimed at achieving socially intended effects (in this case the specialist training of GPs in sufficient numbers). This project report makes a data-driven contribution to gauging the impact on primary care in Germany of support for specialist training.

## 2. Project description

### 2.1. Background and problem definition

Within primary care covered by statutory health insurers there were, as at the end of 2022, some 55,000 persons working in Germany as a whole (not counting paediatricians). This corresponded to a weighted total number for requirements-planning purposes (*Bedarfsplanungsgewichte*) of about 51,000. (This figure also corresponds to the number of *Arztsitze*, which are positions/locations (allocated by the German healthcare system) for physicians, or *volle Versorgungsaufträge*, which are full public-service supply mandates). Doctors undergoing specialist training are not included in this figure. The number of unfilled allocated positions in primary care has seen a sharp increase in recent years, with Germany’s National Association of Statutory Health Insurance Physicians (KBV) recording almost 5,000 opportunities for self-employed, practice-based work (“vacant allocated positions”) nationwide as at the end of 2022. In 19 of the 984 designated geographical divisions – termed “planning areas” – for primary care, the relevant federal-state-level committees had identified an undersupply (as defined by the needs-planning directive of the G-BA [2]), with an impending shortfall in a further 94 areas.

**Figure 1:**
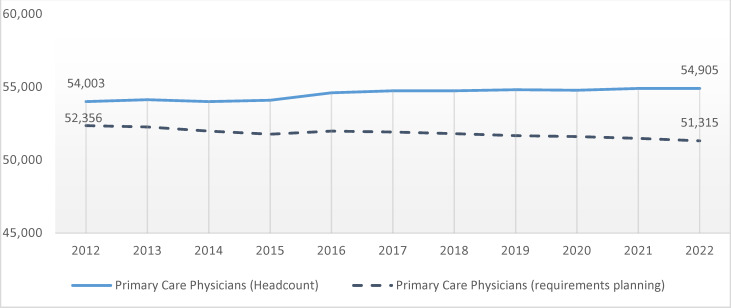
Number of persons and weighted requirements-planning totals in primary care. In 2016, a number of identifiers that had been mis-reported in the past for primary-care and specialist physicians (in the case of internal-medicine specialists) were corrected. The increase for the year 2016 is due chiefly to this correction. Source: German National Register of Physicians (*Bundesarztregister*); SHI-accredited primary-care physicians contributing to healthcare provision (not including paediatricians); all figures as at year’s end.

The age structure in primary care suggests that this trend is set to be exacerbated going forward. As at the end of 2022, the proportion of primary-care physicians aged 60 or over was 36.5%, with 15.7% of them at least 65 years old; the mean age was 55.3 years. Those leaving the primary-care sector are not, in all regions, sufficiently balanced out by newcomers. The vacancy-filling rate, a statistic reflecting the ratio between primary-care doctors entering and leaving the profession, expressed by a weighted requirements-planning total, was below 95% (with 3,048 new starts as against 3,215 departures nationwide). It is striking that, taking Germany as a whole, based on headcount – i.e. without factoring in *Teilnahmeumfang* (literally “extent of contribution”, a measure roughly corresponding to proportion of full-time equivalence (FTE)) – the vacancy-filling rate stood at almost 100% [6]. The total number of persons involved in primary care, therefore, remains virtually constant, but with a decline in total number of physicians providing care at full-scope level (*voller Versorgungsumfang*), and with regional differences. This gradually increasing divergence over time is also evident in the overall number of persons and the weighted requirements-planning total (see figure 1 (Fig. 1)).

In order to address this situation from the supply-side point of view, social-legislation-based support for specialist training was introduced (sec. 75a, SGB V). (This took place alongside, *inter alia*, financial incentives to practise as a specialist in regions affected by – or likely soon to be affected by – undersupply, and the sound integration of general medical practice into foundational student training.) By this means, to ensure sufficient long-term provision of primary and secondary care, both financial and structural support is available for training in outpatient care within one’s chosen speciality. This funding is provided in equal part by regional associations of statutory health insurance (SHI)-accredited physicians (“Kassenärztliche Vereinigungen”, KVen), and by other payers (including health insurers) [4].

This analytical project is aimed at making a data-driven contribution to gauging the impact of such support upon primary care in Germany, while also providing pointers for further specific research. All GPs who became statutory health insurance (SHI)-accredited physicians between 2016 and 2022 were included in the study, both as regards previous support received for specialist training (“Did you participate …? yes/no”) and the nature of their entry into this sector (Teilnahmestatus or “contribution status”, and Teilnahmeumfang or “extent of contribution”). As well as identification of the need for further research, hypotheses on the effects of support arrangements to date are developed. 

### 2.2. Data used and methods

This analysis is based on two data sets which, in combination, allow conclusions to be drawn about the number, and structural aspects, of new GPs (who are support recipients) taking up their first roles. The first data set used is a directory of physicians who have received support for specialist training; the second contains details of practice-based doctors listed in the national registry of physicians (*Bundesarztregister, BAR*) kept by the KBV. The advantages of using these listings are that each of them includes the totality of persons who are support recipients and practise as SHI-accredited physicians, and that – unlike the case with surveyed samples of the overall group, for instance – distortion due to (self-)selection effects is virtually impossible. Possible sources of error are outlined in section 3.3 (“Limitations of the data and possible sources of error”). 

There are two linking elements between the list of support recipients and the national registry of physicians: the AiW number (AiW=*Arzt in Weiterbildung*, i.e. doctor undergoing specialist training) and the lifelong physician number (LANR). When they participate in support, physicians are assigned an AiW number by the KV they belong to. This number is extracted (by the KVen) from the list of numbers that also serves as the source of LANR numbers for SHI-accredited physicians. When, after completing their supported specialist training, doctors start working as SHI-accredited physicians, their AiW number automatically becomes their LANR number. Through cross-checking of AiW numbers (from the list of support recipients) with LANR numbers (from the national registry of physicians), conclusions can be drawn about supported doctors’ patterns of practice-based self-employment and relocation movements. Under the heading “nature of role taken up”, a distinction in status is made between registered (self-employed work), salaried (practice-based or in an ambulatory healthcare centre (MVZ)) and authorized (with this authorization generally awarded to hospital physicians for individual services).

For this purpose, the LANR numbers of new GPs covering the final seven years (2016–2022) were extracted from the national registry of physicians. To count as a new GP, doctors had to have been working as at the end of a given year (but not of the previous year) as a specialist in general practice with the status of an SHI-accredited physician. Based on these criteria, a total of 12,555 persons (7,959 of them female and 4,596 male) were included in the analysis. Data on physicians taken from the list of support recipients was for the support years 2013–2021. The analysis undertaken of the results is purely descriptive in nature. Both the national registry of physicians and the list of support recipients include the totality of persons in the groups investigated, so that the results can be regarded as an exhaustive survey, albeit with the methodological constraints specified in section 3.3. 

## 3. Results

### 3.1. Rate of support uptake, und take-up of roles as SHI-accredited physicians 

To assess the overall impact of support for specialist training, and to gauge how conclusive further studies will be, the authors determined the proportion of new GPs (as listed in the national registry of physicians) who had received support under sec. 75a SGB V. The results are given in table 1 (Tab. 1).

For all of the years under review, a majority of new GPs had received support for specialist training. Averaged over this period, the ratio between receipt of support and practice establishment (*Förder-/Niederlassungsquote*) was equivalent to 71%, i.e. more than two in every three new GPs had previously received financial and structural support with their specialist training. 

Two particularly striking aspects are, firstly, the considerable increase over time in the number of those taking up roles with SHI-accredited status, and secondly, the commensurate rise in the proportion receiving support. Whereas in 2016 just over half (57%) of all new GPs had taken part in support measures for specialist training, by 2022 this was the case for more than four out every five new GPs (81%). 

### 3.2. Patterns in GP practice establishment: structural characteristics 

When looking at new GPs in terms of whether they had or had not received support, one particularly interesting aspect is whether there are structural differences between these two groups. 

As in other professional groups, the increase in uptake of part-time roles (i.e. roles at less than full-scope level) and salaried roles in practices and ambulatory healthcare centres (“contribution status”) are sustained trends that reduce the proportion of those in self-employed and full-time roles.

Data for supported and non-supported new GPs was analysed in terms of these two characteristics. Results for “contribution status” are given in table 2 (Tab. 2), with those for “extent of contribution” presented in table 3 (Tab. 3).

Comparison of supported and non-supported new GPs revealed marked differences as regards the two structural aspects, namely “contribution status” and “extent of contribution”. 

Supported new GPs are considerably more likely to work as self-employed SHI-accredited physicians than are non-supported GPs (38% vs. 30% over the final seven years). What is striking here is that the overall proportion of those in self-employed work declined somewhat over time, whereas the difference between the groups showed an increase. In the final year under review in which practice establishment was included (2022), 33% of supported new GPs were working as self-employed SHI-accredited physicians as opposed to only 21% of those who had not received support. Conversely, the proportion of salaried doctors in ambulatory healthcare centres was far higher among non-supported new GPs than in their supported counterparts (17% vs. 11% over the final seven years under review). By contrast, the proportion taking up salaried, practice-based roles – the option with the highest take-up among new GPs – exhibited virtually no difference when broken down by support status. 

Marked differences are also evident in the extent of doctors’ “contribution” to healthcare provision: of supported new GPs, 66% contributed at full-scope level, whereas for those without support the proportion was only 53%. To put this another way: a new physician who has just entered the healthcare system after receiving support with specialist training is about twice as likely to be working full-time as part-time. By contrast, among non-supported new physicians the proportion of those in full-time and part-time roles is virtually equal.

### 3.3. Limitations of the data and possible sources of error

The data used that was taken from the list of support recipients spans the support years 2013–2021, whereas that for new GPs covers the period 2016–2022. Owing to the duration of specialist training, conclusions about the overall population of support recipients are only indirectly possible based on the data available. This means that particularly “fast-moving” supported doctors, who completed their specialist training during the period under review, are over-represented among those entering the healthcare system as SHI-accredited physicians compared with those gaining their specialist qualification over a longer period. As to whether systematic differences come into play here that affect the analysis performed, it will be possible to draw empirical conclusions on this only after several additional years of support for specialist training have elapsed.

The subject of this study is the totality of those SHI-accredited physicians (as extracted from the national registry of physicians) who have started contributing to healthcare provision, and for whom data cross-checking has ascertained the proportion that had, in the past, undergone support for specialist training. Converse conclusions – i.e. about the proportion of the totality of support recipients who subsequently began working as SHI-accredited physicians – are not possible given the study design used here.

Limitations, where present, may result from any mismatches between AiW number and subsequent LANR number; these can be due to name changes occurring in the interim, errors in data maintenance or possibly specific regional aspects of the respective KVen. Physicians undergoing specialist training who have opted out of the use of their data for research purposes, cannot be identified as support recipients. Where there is a mismatch between LANR and AiW numbers, those who have recently taken up self-employed, practice-based roles cannot be identified as former support recipients. Statistics on supported new GPs should, therefore, be regarded as minimum numbers, with the true figures more likely to be higher than lower.

Not included in records of recipients of support for specialist training, and hence not covered by the present analysis, are professional groups involved in primary care who are not GPs. These groups chiefly comprise internal-medicine specialists providing primary care – who are not the intended recipients of social-legislation-based support for specialist training – and primary physicians without specialized training (*praktische Ärzte*), as these professional designations are no longer awarded. Combined, these two groups account for around one-third of primary care provided: as at the end of 2022, the total of 54,905 primary-care physicians included 17,560 internal-medicine specialists and 3,189 primary physicians without specialized training [6]. By the same token, no conclusions can be drawn about GPs in the private healthcare sector. 

## 4. Discussion

The aim of this analytical project is to highlight potential effects of support for specialist outpatient-care training on healthcare provision by SHI-accredited physicians, and to evaluate this support in its own right. 

Especially with regard to the emerging shortage of doctors in many professional groups, and the growing undersupply in many regions (cf. [1], *inter alia*) hard data is required to inform future policy on structural aspects of outpatient healthcare provided by SHI-accredited physicians. The data drawn upon for this study opens up avenues for further research and investigations in this field.

Studies on foundational and specialist training in medicine – especially those on support for specialist training – mainly involve qualitative research methods. Quantitative surveys in the literature often have a specific regional focus and are based on small sample sizes. Among the main emphases here are the various content-related aspects of specialist training [7] and expectations that the new generation of medical practitioners have as to their future career roles [8], as well as rationales and decision-making processes involved in the choice of speciality to train in [9]. 

More comprehensive surveys include the KarMed study – a prospective, multicentre cohort study exploring career paths taken by physicians during their specialist training – and a careers-monitoring survey of medical students conducted every four years by the University of Trier in conjunction with the KBV, the Medical Faculty Association (MFT) and the German Medical Students’ Association (bvmd). These provide valuable and thought-provoking insights into expectations and desires held by the next generation of doctors, and can provide pointers as to future developments – some of which are already evident in the present analysis [10], [11].

The analysis suggests that doctors who had received support with specialist training go on to become SHI-accredited physicians after having obtained specialist certification as general practitioners. 

It is now the case that a majority of new GPs have undergone social-legislation-based support (under Sec. 75a SGB V) with their specialist training (cf. also section 3.3 on limitations). Given the growing shortage of primary-care physicians, two current trends are particularly welcome: the marked rise in the rate of support uptake, and a considerable increase (in terms of absolute numbers) in those taking up GP roles. 

Further research is needed to explain why support recipients are more likely than their non-supported counterparts to enter the healthcare system as SHI-accredited physicians in full-time and self-employed roles. Career monitoring of medical students [11] showed that respondents do not feel adequately prepared for outpatient care and, in particular, for working in their own practice. The KarMed study came to a similar conclusion: in many different disciplines, doctors doing specialist training felt that what they learned in inpatient medicine does not equip them sufficiently for a role in the outpatient sector [12]. However, support for specialist training may achieve precisely this: prospective specialist physicians are, thanks to close supervision during training in outpatient care within their chosen speciality, well prepared for roles in the outpatient sector and hence more likely to work as SHI-accredited physicians; they may well choose to set up on their own. Furthermore, characteristics of the specialist-training place may be a factor promoting take-up of full-time or self-employed roles: specialist training in an owner-run practice may encourage new doctors to follow suit and take up self-employed, full-time roles, whereas those employed in an ambulatory healthcare centre are less likely to go down this path. This hypothesis, too, requires further academic verification.

The trend emerging towards salaried and part-time roles also applies to physicians in receipt of support for specialist training, but to a different extent than for those without this assistance. In particular with respect to the expectations and desires of future generations in the medical profession – regarding flexible working-hours arrangements, salaried roles and work activities in an interprofessional team with close interdisciplinary interaction [11], [12] – the outcome of this data analysis reflects precisely these current developments. Meeting the need for primary care in Germany will remain a challenge due to the continuing trend towards part-time working.

## 5. Conclusion

This data analysis reveals a marked increase in both the take-up of support and the number of new GPs within the period under review. Although, based on the data, a cause-and-effect relationship cannot be established, the analysis reveals that doctors receiving support for specialist training go on to become SHI-accredited physicians. It appears that support for specialist training may be an instrumental factor here in ensuring provision of primary care, and help combat the shortage of physicians that is emerging and (in many regions) already making itself felt.

Outpatient care – including primary healthcare provision in particular – continues to face major challenges for which timely solutions must be found in order to ensure the medical care needed can continue to be provided throughout Germany. In this, there is a particular need to examine the role played by expectations and wants that future generations of medics hold. Part-time working, the desire for a salaried role rather than setting up on one’s own, and for working in an interprofessional team, are becoming ever more relevant and are set to influence demands vis-à-vis working conditions experienced by SHI-accredited physicians. In the light of such challenges, these results could form a springboard for further research into social-legislation-based support of specialist training, for examining the impacts of support on one’s subsequent work in medicine, and for analysing the effectiveness of incentive models.

The present analysis offers pointers (which themselves require further investigation) as to the effectiveness and sustainability of supported specialist training. The hypothesis that supported specialist training serves as excellent preparation for prospective doctors – so that the concerns identified in the above-mentioned studies can be dispelled – will need to be reviewed by subsequent research.

## Authors’ ORCIDs

- Simon Rass: [0009-0004-4149-071X]
- Charlotte Weber: [0009-0000-2197-0792]
- Bernhard Gibis: [0009-0001-9399-327X]

## Acknowledgements

The authors are grateful to Regina Reuschenberg for contributing her expertise during the preparation of this project report.

## Competing interests

The authors declare that they have no competing interests. 

## Figures and Tables

**Table 1:**
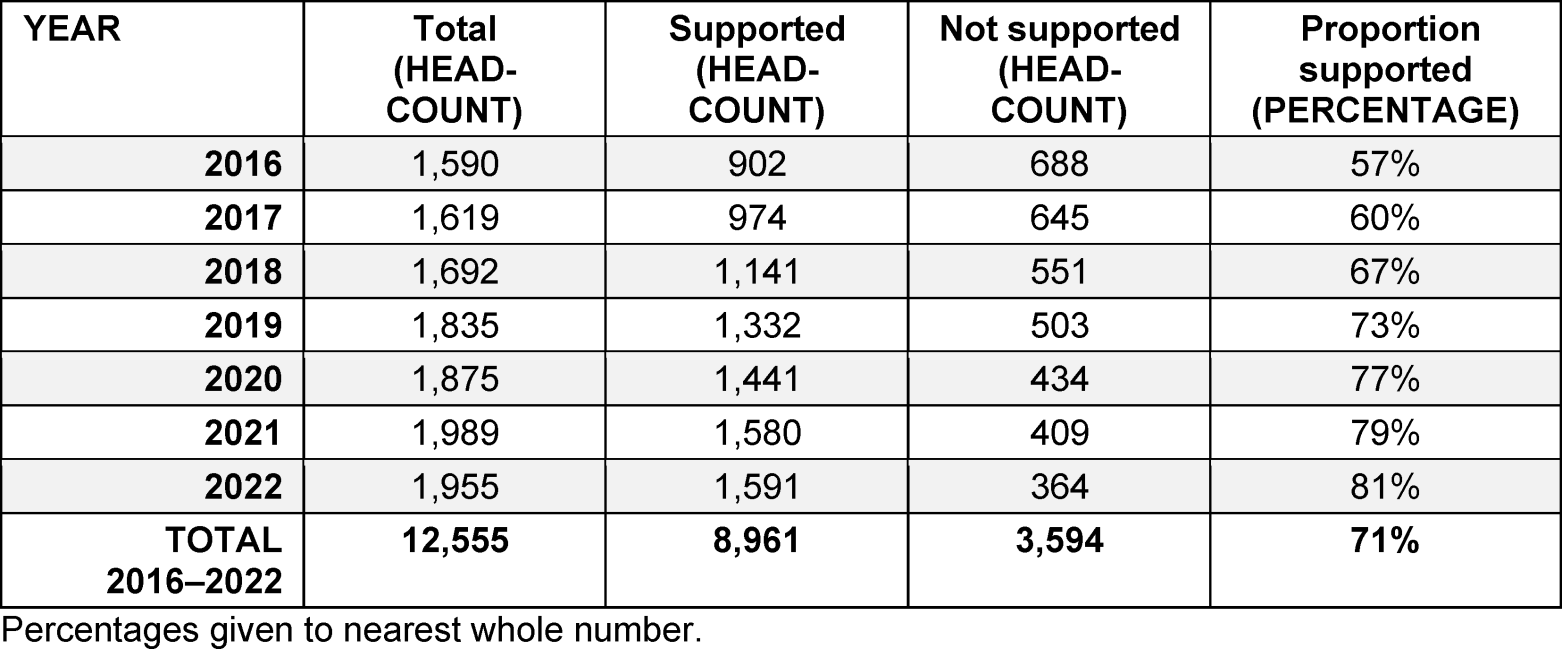
Rate of support take-up over the period 2016–2022 by new, SHI-accredited general practitioners

**Table 2:**
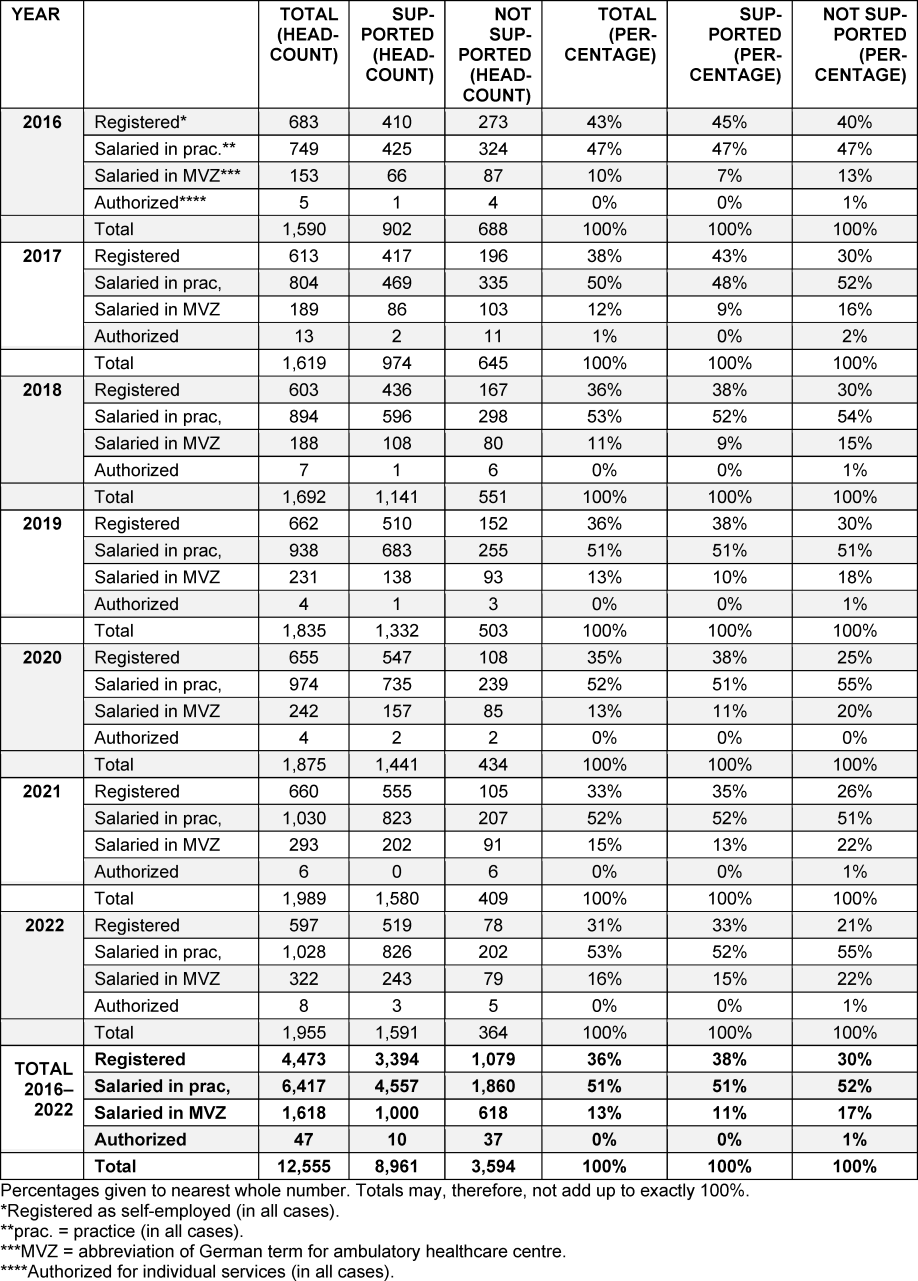
New SHI-accredited general practitioners who began working in the years 2016–2022, broken down by support status (specialist training supported/not supported) and “contribution status” when they took up their first role (registered/salaried/authorized)

**Table 3:**
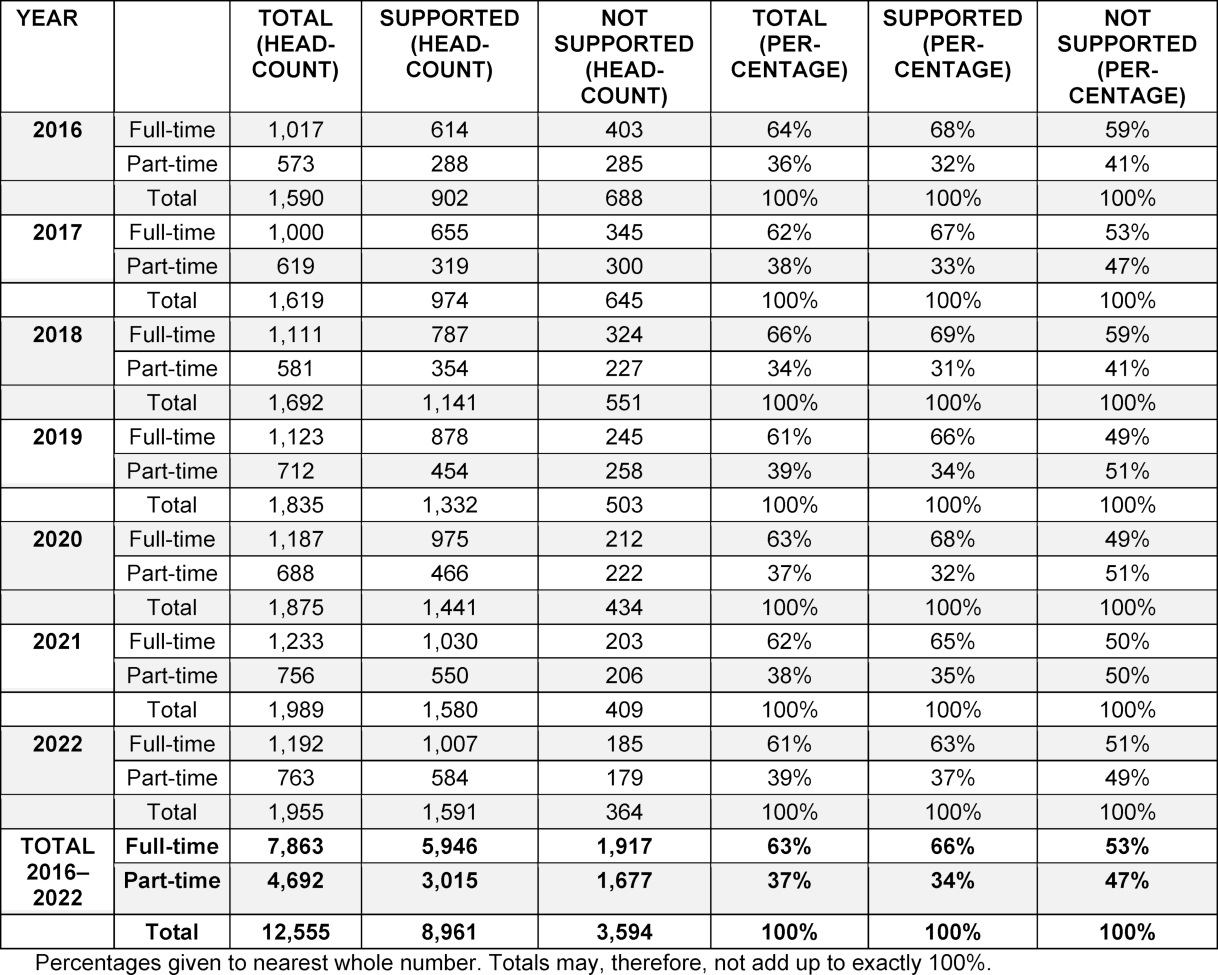
New SHI-accredited general practitioners who began working in the years 2016–2022, broken down by support status (specialist training supported/not supported) and “extent of contribution” when they took up their first role (full-time/part-time)
